# Ozone Therapy and Negative Pressure Wound Therapy in the Treatment of Difficult-to-Heal Wounds in Diabetic Foot Syndrome and Charcot Neuroarthropathy

**DOI:** 10.3390/jcm14124017

**Published:** 2025-06-06

**Authors:** Agnieszka Białomyzy, Katarzyna Kotrych, Anna Bogacz, Marta Podralska, Aleksandra Górska, Jacek Białecki, Izabela Uzar, Bogusław Czerny, Adam Kamiński

**Affiliations:** 1 Eter Med Medical Center Gdansk, Żabi Kruk 10, 80-822 Gdansk, Poland; agnieszka.bialomyzy@gmail.com; 2 Department and Clinic of General Endocrine Surgery and Gastrointestinal Oncology, Poznan University of Medical Sciences, Przybyszewskiego 49, 60-355 Poznan, Poland; 3 Department of General and Dental Radiology, Pomeranian Medical University in Szczecin, al. Powstańców Wielkopolskch 72, 70-111 Szczecin, Poland; kotrych1@gmail.com; 4 Department of Physiology, Poznan University of Medical Sciences, Smoluchowskiego 11, 60-179 Poznan, Poland; 5 Department of Stem Cells and Regenerative Medicine, Institute of Natural Fibres and Medicinal Plants, Kolejowa 2, 62-064 Plewiska, Poland; marta.podralska@iwnirz.pl (M.P.); aleksandra.gorska@iwnirz.pl (A.G.); bczerny@wp.pl (B.C.); 6 Department of General Minimally Invasive and Trauma Surgery, Francis Raszeja Municipal Hospital, Mickiewicza 2, 60-834 Poznan, Poland; jacekt.bialecki@gmail.com; 7 Department of Pharmacology and Pharmacoeconomics, Pomeranian Medical University in Szczecin, 71-230 Szczecin, Poland; uzari@wp.pl; 8 Department of Children Orthopedics and Musculosceletal Oncology, Pomeranian Medical University in Szczecin, Unii Lubelskiej 1, 71-252 Szczecin, Poland; emluc@wp.pl

**Keywords:** Charcot neuroarthropathy, diabetic foot syndrome, negative pressure wound therapy, ozone therapy

## Abstract

Diabetes, as one of the most common diseases of civilization, is a significant factor of mortality worldwide. Undiagnosed and improperly treated, it leads to the development of a number of complications, including diabetic foot syndrome (DFS) and Charcot neuroarthropathy (CN). Charcot neuroarthropathy is a complex and devastating disease characterized by the presence of neuropathy, progressive deformities, and joint destruction. Risk factors and epidemiological data emphasize the high prevalence of CN in the diabetic population, drawing attention to typical predisposing factors for the development of this disease. Serious complications, such as foot ulcers or amputations, show the scale of the negative impact of CN and DFS on the quality of life of patients. **Background/Objectives**: The aim of the study was to assess the treatment of foot ulcers in patients with DFS and CN using ozone therapy with simultaneous negative pressure wound therapy (NPWT). **Methods**: The study included 30 patients aged 39 to 87 years with DFS and 30 patients with CN. Ozone therapy and negative pressure wound therapy were used for the treatment of chronic wounds. **Results**: The analysis of the results showed a significant reduction in the wound size in both study groups; in patients with DFS, a reduction from 5 cm^3^ to 0.40 cm^3^ observed after 3 weeks and to 0.002 cm^3^ after 6 weeks of therapy, while in patients with CN, a reduction from 8 cm^3^ to 1.50 cm^3^ was observed after 3 weeks and to 0.004 cm^3^ after 6 weeks of therapy. No statistically significant differences were observed in median wound sizes between the DFS and CN groups. Ozone therapy with a value of 70 μg/mL is an effective method in the treatment of chronic diseases of soft tissue and the skeletal system. In combination with NPWT after cleansing the wound of bone sequestrum, the process increased the density of capillaries by accelerating the synthesis of proteins and collagen and reduced bacterial colonization in the wound. **Conclusions:** The use of ozone therapy procedures at 70 μg/mL with negative pressure therapy is effective in the prevention and treatment of infectious bone complications in diabetes, such as diabetic foot syndrome and Charcot neuroarthropathy.

## 1. Introduction

Diabetes mellitus (DM) constitutes a heterogeneous group of metabolic disorders characterized primarily by persistent hyperglycemia. This condition arises from either an absolute or relative deficiency of insulin, resulting in widespread disturbances of cellular metabolism and culminating in multi-organ dysfunction [1]. According to the 2021 report by the International Diabetes Federation (IDF), approximately 537 million individuals globally are affected by diabetes [2,3]. Moreover, estimates from the World Health Organization (WHO) suggest that the current global prevalence of diabetes cases exceeds 830 million [4,5]. Poorly controlled diabetes significantly predisposes patients to numerous complications, notably diabetic foot syndrome (DFS), which can lead to profound disability and increased mortality risk. The WHO defines DFS as infection, ulceration, or destruction of deep tissues of the foot, including bone, resulting from varying degrees of peripheral neuropathy and vascular disease [6]. The annual incidence of DFS is estimated at approximately 2% of the general population [7,8], while the lifetime risk of developing DFS among diabetic patients ranges between 19% and 34% [7]. Consequently, the risk of ulcer formation in individuals with diabetes is substantially elevated, approximating 25% [9].

Management of diabetic foot ulcers remains a significant clinical challenge. Minimally invasive surgical interventions have emerged as pivotal strategies to promote healing and functional recovery in patients with DFS. Distal metatarsal diaphyseal osteotomy (DMDO), involving a distal osteotomy of the proximal metatarsal neck, aims to alleviate pressure on ulcerated areas and expedite tissue repair. Preliminary prospective studies have demonstrated DMDO to be a safe and efficacious technique facilitating healing of chronic diabetic foot ulcers across various stages of severity [10,11].

Charcot neuroarthropathy (CN) represents a chronic, progressive, and debilitating pathology primarily affecting the osseous and articular structures of individuals with peripheral neuropathy, most frequently secondary to diabetes mellitus. This condition is typified by either painful or insensate destruction of bones and joints in limbs deprived of protective sensory innervation [12,13]. Epidemiological investigations and risk factor analyses reveal a high prevalence of CN among diabetic populations, with critical determinants including glycemic control, disease duration, and diabetes subtype playing integral roles in risk stratification. CN is associated with severe sequelae such as foot ulceration and limb amputation, substantially impairing patients’ quality of life [14,15].

Osteitis in the context of DFS and CN poses significant diagnostic and therapeutic challenges. Selection of appropriate treatment modalities should be guided by a comprehensive assessment of the patient’s clinical status, supported by laboratory, microbiological, and imaging data. It is essential to differentiate neuropathic alterations from active osteomyelitic inflammation to tailor therapeutic interventions effectively. Prompt initiation of innovative and targeted therapies is critical, as delays may precipitate irreversible tissue damage and necessitate amputation.

The primary objective of the present study is to assess the efficacy of adjunctive ozone therapy combined with negative pressure wound therapy (NPWT) in the management of foot ulcers among patients diagnosed with DFS and CN.

## 2. Materials and Methods

### 2.1. Study Group

The retrospective study included 30 patients with diabetic foot syndrome (DFS) and 30 patients with Charcot neuroarthropathy. In both cases, the disease process was characterized by infection of soft tissues and bones located in the toes, forefoot (in DFS), and midfoot on the plantar side of the foot (in CN). A total of 60 patients treated at the Eter-Med Health Centre—Chronic Wound Treatment in Gdańsk between 2019 and 2022 were included in the study. The opinion of the Bioethics Committee of the Pomeranian Medical University in Szczecin was obtained (no. KB.006.130.2023). Patients were included in the retrospective analysis if they met specific clinical and diagnostic criteria. The study population comprised individuals diagnosed with diabetic foot syndrome (DFS) or Charcot neuroarthropathy (CN) presenting with chronic foot ulcers and clinically and radiologically confirmed soft tissue infection accompanied by bone involvement (osteitis/osteomyelitis). Eligible participants had previously undergone conservative treatment, including the use of advanced wound dressings and symptomatic therapy, which proved ineffective. Inclusion in the DFS group required the presence of chronic ulceration involving the toes or forefoot, with soft tissue and bone infection confirmed by imaging (osteitis/osteomyelitis) and no structural midfoot deformity. The CN group assignment was based on the presence of plantar midfoot ulceration, typical radiographic features of Charcot neuroarthropathy (e.g., joint disintegration and midfoot collapse), and concurrent bone infection. Patients were excluded from the study if they met any of the following criteria: absence of radiological evidence of bone infection, the presence of severe comorbidities contraindicating the use of ozone therapy or NPWT, or coagulation disorders and other medical conditions precluding surgical intervention at the wound site. Clinical data and an individual algorithm for the treatment of soft tissue and bone inflammation in wounds occurring in DFS and CN were developed. A survey was conducted among all study participants to assess the wound in DFS and CN after 3 and 6 weeks of treatment. The study included 45 men (75.0%) and 15 women (25.0%). The age of the subjects ranged from 39 to 87 years (mean age 66.68 ± 13.40 years). The treatment so far included symptomatic treatment and the use of specialist dressings, which did not have a positive therapeutic effect. In both disease entities, laboratory and bacteriological tests were performed. The patients underwent laboratory tests to assess their general condition and active inflammatory process, suggesting bacterial colonization of the wounds. The tests mainly included determination of the level of glycated hemoglobin, CRP, and procalcitonin.

The ankle–brachial index (ABI) was used to assess the condition of peripheral arteries. The ABI was determined separately for the right and left limbs of the patient. The final result for the patient was the ABI with the lower value. Transcutaneous oxygen tension (tcpO_2_) was performed. To obtain a complete clinical picture, Doppler ultrasound of the veins was performed using the GE Versona Primer ultrasound device (Sacramento, CA, USA). Radiological examinations revealed osteitis in both groups. In all patients, dead bone fragments were eliminated to the border of the bleeding bone and the surrounding healthy tissue. The group of patients underwent a 6-week period of specialist treatment until the epithelialization stage was reached. In 30 patients with diabetic foot ulcers, the wounds healed completely without recurrence. In the group of patients with CN, the wound healed in 20 patients, and in 10 patients, local superinfection of the epithelialization process occurred. They underwent further treatment.

### 2.2. Study Design

The study methodology encompassed a comprehensive clinical assessment, including patient interviews and detailed review of prior medical records, complemented by recommended laboratory and microbiological investigations. Vascular status was evaluated using the ankle–brachial index (ABI) and transcutaneous partial oxygen pressure (TcPO2) measurements in diabetic patients. Radiographic imaging, specifically anteroposterior (AP) and lateral X-rays, was utilized to confirm the presence of osteomyelitis. Soft tissue, arterial, and venous evaluations were conducted via Doppler ultrasound (USG) to identify vascular insufficiency. Wound assessment in patients with Charcot neuroarthropathy (CN) and diabetic foot syndrome (DFS) was further classified according to the Wagner grading system.

**Figure 1:**
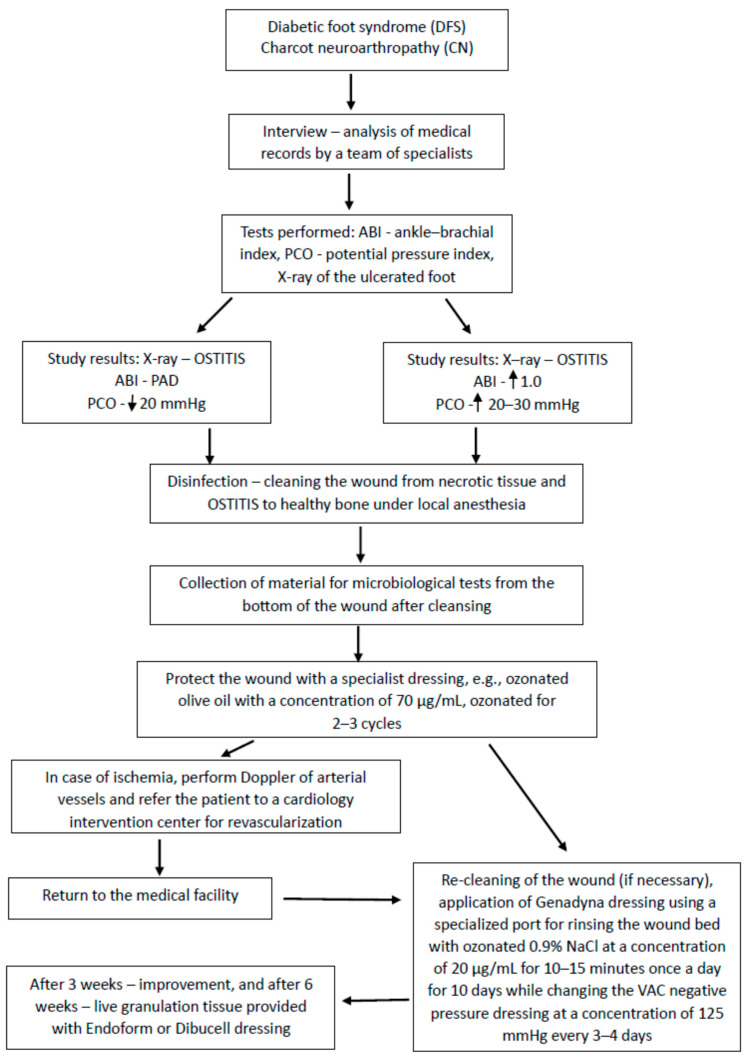
Algorithm of treatment procedure.

**Figure 2:**
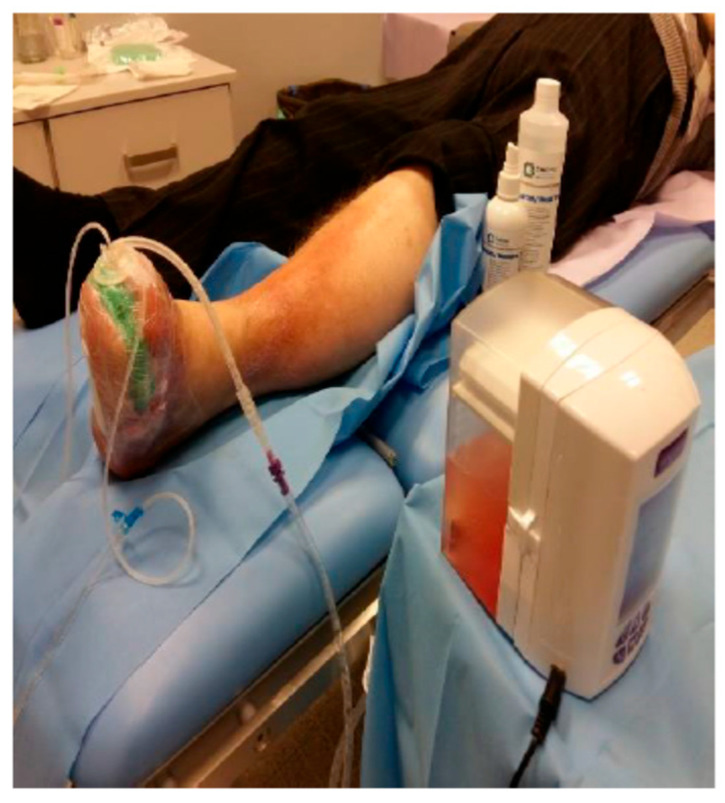
Ozone therapy (ATO3 device) with simultaneous negative pressure wound therapy.

An original therapeutic algorithm was developed, which yielded favorable clinical outcomes within the studied cohort (Figure 1). In addition to the application of antiseptics, targeted antibiotic regimens, and surgical correction of osseous defects, the treatment protocol incorporated ozone therapy (OT) concurrently with negative pressure wound therapy (NPWT) (Figure 2), modalities whose efficacy was substantiated in this context. The investigation compared ozone delivery through three distinct methods: wound irrigation with ozonated distilled water, ozonated 0.9% sodium chloride solution, and administration of an ozone–oxygen gaseous mixture. All modalities employed an ozone concentration of 70 μg/mL, a dosage previously demonstrated to exert potent bactericidal activity.

A comparable analysis was conducted focusing on regenerative parameters associated with negative pressure wound therapy (NPWT), specifically within the range of 80–125 mmHg, in conjunction with simultaneous ozone therapy (OT) utilizing the port device designed by Professor Tomasz Banasiewicz.

### 2.3. Negative Pressure Wound Therapy (NPWT) with Ozone-Enhanced Irrigation

The NPWT system integrated with concurrent ozone-enhanced irrigation employed ozonated 0.9% sodium chloride solution at a concentration of 70 μg/mL, delivered within a closed negative pressure circuit. The treatment protocol comprised three distinct phases:

Phase One: Administration of 500 mL of ozonated 0.9% NaCl (70 μg/mL) generated by an ozone generator, introduced via the port drainage system to saturate the polyurethane sponge dressing adjacent to the wound for 30 min.

Phase Two: Maintenance of the ozonated fluid within the wound environment under negative pressure for a duration of 5 min.

Phase Three: Evacuation of the rinsing fluid from the wound bed.

Each complete cycle lasted approximately 15 min. The duration of fluid retention within the wound varied from 1 s up to 30 min. It was observed that prolonged retention of the ozonated saline enhanced the efficacy of cleansing both the polyurethane interface and the wound surface, with an optimal bactericidal effect achieved after approximately 15 min of ozone exposure.

Occasionally, the second phase was omitted, resulting in a continuous irrigation system, which demonstrated favorable clinical outcomes in the therapeutic response. Negative pressure was typically maintained between 100 and 125 mmHg; pressures below this range were found not to enhance therapeutic efficacy and could potentially impair tissue perfusion, thereby increasing the risk of ischemic injury. The treatment cycle was administered once daily for a duration of 10 days, after which NPWT was continued based on clinical indications.

Following this regimen, sterile bacteriological cultures were consistently obtained. Innovations in the design of the port, combined with the synergistic application of NPWT and OT, facilitated the development of wound beds characterized by robust granulation tissue and closure of fistulous tracts. The volume of irrigation fluid, duration of retention, and applied negative pressure parameters were tailored individually to each patient’s clinical status.

In managing osteomyelitic complications in diabetic foot syndrome (DFS) and Charcot neuroarthropathy (CN), a multimodal therapeutic approach was employed, integrating surgical intervention, antiseptic protocols, analgesia, targeted antibiotic therapy, OT, and NPWT. This integrative strategy leveraged the complementary mechanisms of each modality, culminating in marked reductions in inflammation and promotion of tissue regeneration and wound healing.

Pain intensity was quantitatively assessed before, during, and upon completion of treatment using the Visual Analogue Scale (VAS). This metric enabled standardized comparison of pain levels both intra-individually and between the DFS and CN patient cohorts. At baseline, patients with DFS and bone infection reported severe pain levels ranging from 8 to 10 cm on the VAS. Comparable pain intensities were documented in the CN cohort. After three weeks of therapy, pain scores decreased to 5–8 cm in the DFS group and 6–8 cm in the CN group. By the conclusion of treatment, VAS scores reached 0 in both cohorts, indicating complete resolution of pain.

### 2.4. Statistical Analysis

Statistical analysis of the results was performed using the R software (R Core Team, 2021) version 4.2.2. The normality of the data distribution on an interval scale was assessed using the Shapiro–Wilk test. Normally distributed variables were presented as arithmetic means with standard deviation (±SD), and in the case of non-normality, the distribution was presented as medians with lower and upper quartiles (Q1 and Q3). To assess the difference in the range of compared parameters between groups, when a normal distribution was obtained, the Student’s *t* test was used, and for nonparametric data, the Mann–Whitney U test was used. Categorical variables presented as numbers and percentages were compared using the χ2 test or Fisher’s test, depending on the size of the compared groups. Friedman’s ANOVA and the Wilcoxon signed-rank test were used to analyze dependent data.

## 3. Results

### 3.1. Characteristics of the Studied Patients

Table 1 shows the general characteristics of patients with DFS and CN. In both groups, the largest proportion of patients were men (80% and 70% in DFS and CN, respectively; *p* = 0.551). There were no significant differences between the groups in the mean values of age, body weight, height, BMI, and the number of cases of smoking cigarettes and drinking alcohol. Patients with DFS and CN suffered from type 1 diabetes (83.33% and 73.33%, respectively; *p* = 0.531). Almost all of the examined patients had limited mobility and used assistance during moving. Patients used crutches (83.33% with DFS vs. 73.33% with CN), walkers (10.00% DFS and 6.67% CN), and wheelchairs (20.00% with DFS vs. 23.33% with CN). Moreover, 20 patients with DFS wore off-loading footwear (66.67%).

### 3.2. Analysis of Laboratory Data

Subsequently, comprehensive laboratory parameters were collected, including biochemical profiles, complete blood counts, and coagulation indices. Glycated hemoglobin (HbA1c) levels were comparable between both cohorts, with median values of 7.20%, and no statistically significant difference was observed (*p* = 0.689). Notably, hemoglobin (HGB) concentrations were significantly elevated in patients with diabetic foot syndrome (DFS) compared to those with Charcot neuroarthropathy (CN) (12.59 ± 1.89 g/dL vs. 11.09 ± 1.46 g/dL, *p* = 0.001).

Conversely, plasma D-dimer concentrations were significantly higher in the CN group, with median values of 484.00 µg/L (IQR: 420.00–500.00) compared to 400.00 µg/L (IQR: 280.00–470.00) in DFS patients (*p* = 0.004). Additionally, a trend towards elevated serum potassium levels was observed in CN patients (4.71 ± 0.57 mmol/L) relative to the DFS group (4.46 ± 0.38 mmol/L), approaching statistical significance (*p* = 0.051). Uric acid levels were also increased in the CN cohort (median 5.00 mg/dL, IQR: 4.00–6.00) compared to DFS patients (4.00 mg/dL, IQR: 3.00–5.00), although this difference did not reach statistical significance (*p* = 0.064). No statistically significant intergroup differences were detected in fibrinogen levels, activated partial thromboplastin time (APTT), protein C activity, serum sodium, or total protein concentrations (Table 2).

Results are presented as medians [lower and upper quartile] and arithmetic means and standard deviations.

Inflammatory markers, specifically C-reactive protein (CRP) and procalcitonin (PCT), were assessed longitudinally at baseline (enrollment) and at subsequent intervals of 3 and 6 weeks post-initiation of therapy (Table 3).

At baseline, the DFS cohort exhibited higher median CRP values (195.50 mg/L, IQR: 110.00–280.00) relative to the CN group (122.50 mg/L, IQR: 80.00–220.00), although this difference was not statistically significant (*p* = 0.087). Procalcitonin levels were significantly elevated in DFS patients at baseline (median 0.86%, IQR: 0.64–0.96) compared to CN patients (median 0.61%, IQR: 0.46–0.76; *p* = 0.003). This significant elevation in PCT persisted in the DFS group at both 3 weeks (*p* = 0.003) and 6 weeks (*p* = 0.015) of therapy. Furthermore, CRP levels measured at 6 weeks post-treatment were significantly higher in DFS patients (median 8.00 mg/L, IQR: 5.00–10.00) than in the CN group (median 5.00 mg/L, IQR: 5.00–6.00; *p* = 0.002) (Table 3). Despite these intergroup differences, both CRP and PCT concentrations demonstrated statistically significant reductions over the course of follow-up within each group (*p* < 0.001).

### 3.3. Analysis of Treatment Procedure

Ozone therapy (OT) in conjunction with negative pressure wound therapy (NPWT) was implemented for the management of chronic wounds at the Enter-Med clinical center. Initial treatment protocols for all patients involved meticulous wound cleansing and preparation, including the use of antibacterial soap for local toileting, irrigation with ozonated distilled water at a concentration of 70 µg/mL, followed by disinfection with Microdacyn^®^ solution, which contains sodium hypochlorite and hypochlorous acid and exhibits potent local antimicrobial properties. Surgical debridement was routinely performed to excise necrotic tissue and devitalized bone fragments, thereby optimizing the wound bed for healing.

Among the cohort, no patients presented with lesions necessitating immediate amputation. However, within the diabetic foot syndrome (DFS) subgroup, 8 individuals were identified as having an elevated risk for amputation of the hallux, and 10 patients exhibited risk factors for amputation involving multiple digits. Conversely, only one patient in the Charcot neuroarthropathy (CN) group was categorized as being at risk for toe amputation.

The frequency and nature of therapeutic interventions administered to patients with DFS and CN are summarized in Table 4. Comparative analysis revealed a higher, albeit statistically nonsignificant, utilization of bone graft substitutes, specifically beta-tricalcium phosphate (Beta-TCP), among CN patients (16.67%) relative to the DFS group (10.00%, *p* = 0.704). Additionally, vacuum-assisted closure was employed more frequently in the CN cohort (70.00%) compared to DFS patients (46.67%, *p* = 0.116). In contrast, the application of specialized ozonated oil dressings was significantly more prevalent in the DFS group, with universal use (100.00%) compared to 50.00% in the CN group (*p* < 0.001).

### 3.4. Assessment of the Blood Vessels’ Condition

Vascular insufficiency causes tissue hypoxia, which affects the wound healing process. The ankle–brachial index (ABI) was used to assess the condition of peripheral arteries. ABI was determined separately for the right and left limbs. The final result was the calculated ABI from the measurement with the lower value. No differences in the median ABI values were observed between the analyzed groups (*p* = 0.176). Transcutaneous oxygen tension (tcpO_2_) was measured using the MEDICAP Precise 8001 device. A statistically significantly lower value of tcpO_2_ was noted in CN patients (median of 35.00 mmHg [Q1—30.00; Q3—50.00]) compared with the DFS group (median of 45.00 mmHg [Q1—35.00; Q3—60.00]), (*p* = 0.018). Doppler USG of the veins was also performed using the GE Versona Primer USG device. Thrombosis was diagnosed in 16.67% of patients with DFS and 30.00% of patients with CN (*p* = 0.360). Lower limb ischemia occurred in 13.33% of patients with DFS and 20.00% of patients with CN (Table 5).

### 3.5. Wound Assessment

Each patient underwent wound assessment before and after 3 and 6 weeks of treatment to monitor the healing process and treatment effects. The results are summarized in Table 6. A significant reduction of wound size was observed in both treatment groups when comparing the wound volume at the final assessment with the baseline value. In the DFS group, the median wound volume at baseline was 5 cm^3^ (IQR: 2–32 cm^3^), which decreased to 0.40 cm^3^ after 3 weeks and to 0.002 cm^3^ after 6 weeks. In the CN group, the baseline wound volume was 8.00 cm^3^ (IQR: 2.40–19.20 cm^3^), which decreased to 1.50 cm^3^ after 3 weeks and to 0.004 cm^3^ after 6 weeks. No statistically significant differences were observed in wound size between the DFS and CN groups. The median wound reduction after 3 weeks of treatment was statistically significantly greater in the DFS group (92.71%; IQR: 75.00–96.25%) compared to the CN group (76.98%; IQR: 62.50–91.11%) (*p* = 0.005). After 6 weeks of therapy, the wound size in patients with DFS was reduced by 99.98% and in patients with CN by 99.90% (*p* = 0.055) (Table 6).

### 3.6. Ulcer Assessment

All study participants underwent foot ulcer assessment immediately after presentation to the clinic in order to develop an appropriate treatment plan. Statistically significant differences between the analyzed groups concerned the duration of the ulcer and their previous treatment. The locations of wounds in patients from the studied groups were also compared. The wounds in all 30 people with CN were located on the sole of the foot (100%) and in 6 people (20%) on the forefoot. In diabetic foot syndrome, ulcers occurred most often on toe I (60%), toes II–V (57%), the sole (47%), and the forefoot (23%) (Table 7).

In order to characterize the diabetic foot more precisely, the Wagner classification was used. One person (3%) with ulceration and inflammation of the skin and subcutaneous tissues was classified as grade 2. Most often, the ulcers were deep, and most patients were classified as grade 3 with advanced changes involving bone structures and foot phlegmon (17 patients—57%). In addition, 9 patients (30%) were classified as grade 4 (local dry necrosis treated conservatively or gangrene of the toes), and 3 people (10%) were classified as grade 5 with extensive necrotic change with an indication for amputation. After three weeks of treatment, most people (21 patients, 70.00%) were classified as grade 2, 8 patients (26.67%) as grade 1, and 1 patient (3.33%) as grade 3. After six weeks of treatment, 16 people (53.33%) were classified as grade 1 (superficial ulceration) and 14 people (46.67%) as grade 0 (deformed foot without ulceration).

### 3.7. Wound Infection

The presence of pathogenic bacteria within the wound environment is a key etiological factor in the development of infection. In both patient cohorts, clinical manifestations of infection—both localized and systemic—were frequently observed. However, patients with diabetic foot syndrome (DFS) demonstrated a significantly higher prevalence of several infection-related symptoms compared to those with Charcot neuroarthropathy (CN). Specifically, increased pain (90.00% vs. 63.33%, *p* = 0.033), erythema (83.33% vs. 33.33%, *p* < 0.001), granulation tissue with visible biofilm puncta (93.33% vs. 70.00%, *p* = 0.045), purulent exudate (80.00% vs. 36.67%, *p* = 0.002), and clinical signs of osteomyelitis (96.67% vs. 56.67%, *p* = 0.001) were significantly more frequent in the DFS group. A comprehensive comparison of the clinical infection parameters at baseline between DFS and CN patients is presented in Table 8.

Microbiological analysis was performed at three time points for each participant: (1) following debridement and removal of necrotic tissue and bone fragments; (2) after 3 weeks of therapy; and (3) at 6 weeks of treatment. At baseline, wounds in both groups were colonized by both Gram-positive and Gram-negative organisms. The most frequently isolated pathogens were *Pseudomonas aeruginosa* (92%), predominantly from bone tissue; *Enterococcus faecalis* (90%); *Staphylococcus aureus* (65%); and *Proteus mirabilis* (27%). No statistically significant differences were found in the prevalence of specific microorganisms between the two groups at baseline.

After three weeks of therapy, radiological examination showed that post-debridement bone structures in both groups were free of new inflammatory foci and exhibited adequate vascularization without signs of ongoing infection. Microbiological cultures at this time revealed persistent P. aeruginosa in 28 patients with DFS (93.3%) and in 26 patients with CN (86.7%) (*p* = 0.667). *S. aureus* was identified in 8 patients with DFS (26.7%) and 11 with CN (36.7%) (*p* = 0.579), while *E. faecalis* was isolated in 5 DFS patients (16.7%) and 4 CN patients (13.3%) (*p* = 1.000). Additional isolates in the DFS group included Streptococcus agalactiae and *P. mirabilis* in single patients.

By week six, only physiological skin flora was detected in 30 DFS patients (100%) and 26 CN patients (86.7%). Residual colonization in the CN group included *S. aureus* in 2 cases (6.7%) and isolated occurrences of *E. faecalis* and *Escherichia coli*.

Antibiotic therapy was implemented in 34 patients (17 DFS and 17 CN) based on microbiological findings. No significant intergroup differences were observed in antibiotic selection. Among DFS patients, the most commonly prescribed antibiotics included amoxicillin (23.33%), clindamycin (10.00%), trimethoprim/sulfamethoxazole (10.00%), ciprofloxacin (6.66%), levofloxacin (6.66%), gentamicin (3.33%), and cefuroxime (3.33%). In the CN group, prescribed agents included trimethoprim/sulfamethoxazole (23.33%), clindamycin (10.00%), gentamicin (10.00%), amoxicillin (6.66%), and cefuroxime (3.33%).

Ozone therapy was employed as an adjunct or alternative to antibiotic treatment, particularly in patients with low total bacterial loads or elevated counts of P. aeruginosa. All patients received wound irrigation with ozonated distilled water (70 µg/mL) and topical application of ozone via a sealed ozone bottle at the same concentration. A specialized dressing impregnated with ozonated olive oil was applied in 28 DFS patients (93.33%), with four treatment cycles in most cases and five cycles in 2 patients (6.67%). Among CN patients, 21 (70.00%) received this form of topical therapy, with four cycles in 15 patients (50.00%) and three cycles in 6 patients (20.00%). The difference in the frequency of ozonated dressing application between the groups was statistically significant (*p* < 0.001).

### 3.8. Ozone Therapy with Simultaneous Negative Pressure Wound Therapy

**Figure 3:**
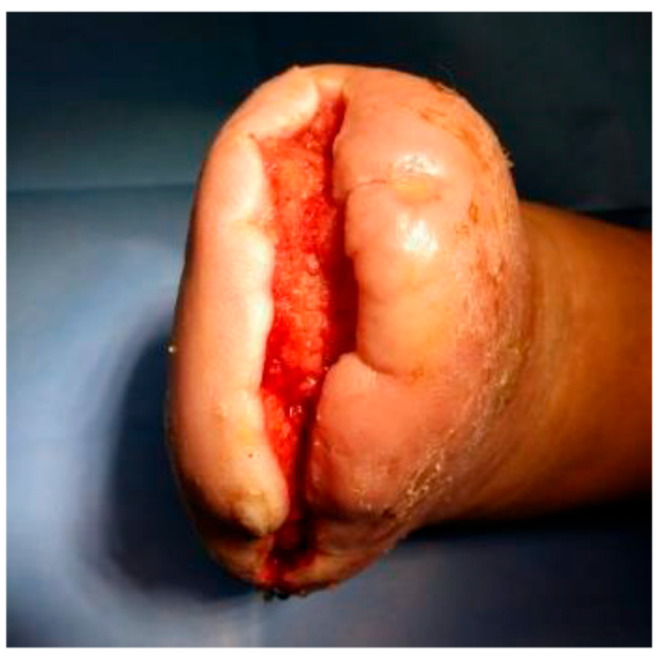
Wound after 4 cycles of ozone therapy with simultaneous negative pressure wound therapy.

**Figure 4:**
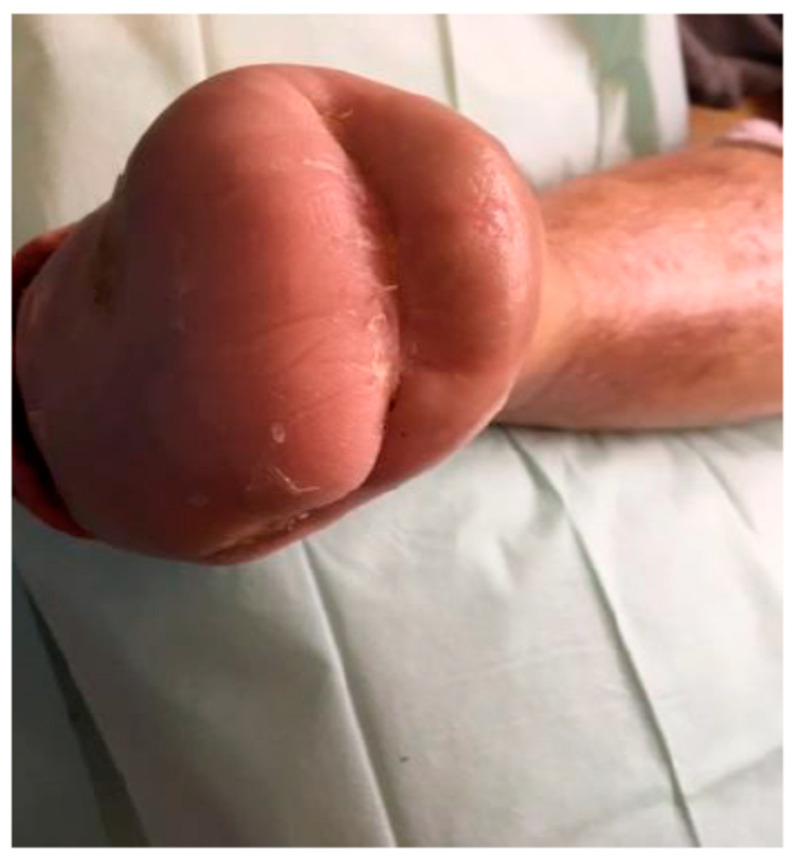
Wound after 15 cycles of ozone therapy with simultaneous negative pressure wound therapy.

Ozone therapy was concurrently administered with negative pressure wound therapy (NPWT) utilizing the Genadyne system in combination with a specialized port developed by Professor Tomasz Banasiewicz (Figure 3 and Figure 4). This combined therapeutic approach was implemented in 39 (65.00%) of the study participants and conducted in four distinct phases. In 12 patients with diabetic foot syndrome (DFS) (40.00%) and 9 with Charcot neuroarthropathy (CN) (30.00%), the second phase—characterized by maintaining wound fluid under sustained negative pressure for five minutes—was omitted (*p* = 0.588).

Throughout the treatment of both DFS and CN, the most commonly applied negative pressure level was −125 mmHg, utilized in 66.67% of DFS cases and 51.72% of CN cases. Wound irrigation with ozonated rinsing solution (70 μg/mL) was performed within the NPWT system either daily for ten consecutive days (in 36.67% of DFS and 60.00% of CN patients) or on alternate days (in 63.33% of DFS and 40.00% of CN patients; *p* = 0.121). The NPWT dressings were most frequently changed every three days (63.33% of DFS and 76.67% of CN cases). In 33.33% of DFS and 23.33% of CN cases, the dressing was changed every four days, while in one DFS patient (3.33%), changes were performed every five days (*p* = 0.385).

Following three weeks of therapy, both patient groups exhibited well-insulated feet with a healthy pink coloration, and resected bone surfaces demonstrated adequate perfusion and absence of inflammatory signs. Capillary density increased by over 200%. Moreover, enhanced cellular proliferation and upregulation of protein and collagen synthesis were observed, contributing to reduced exudate production and a significant increase in viable granulation tissue volume.

After six weeks of treatment, wounds had progressed to the epithelialization phase. At this point, ozone therapy was discontinued, and epithelial coverage was further supported using Endoform or Dibucell biological dressings. All patients achieved independent ambulation without the use of orthopedic aids, requiring only specialized therapeutic footwear tailored to the clinical characteristics of DFS and CN.

## 4. Discussion

The management of chronic, refractory wounds, exemplified by diabetic foot syndrome (DFS) and Charcot neuroarthropathy (CN), constitutes a significant clinical challenge attributable to the multifactorial pathogenesis involving infection, ischemia, neuropathy, and impaired tissue regenerative capacity. Conventional wound care techniques frequently yield suboptimal outcomes, thereby necessitating the integration of advanced therapeutic modalities such as negative pressure wound therapy (NPWT) and ozone therapy. The present investigation, contextualized within the extant scientific literature, delineates the distinct pathophysiological mechanisms inherent to DFS and CN wounds and evaluates the therapeutic benefits of combined NPWT and ozone therapy.

NPWT represents a well-established mechanotherapeutic intervention that expedites wound healing through the application of controlled sub-atmospheric pressure, which facilitates exudate removal, reduces local microbial load, enhances microvascular perfusion, and stimulates granulation tissue proliferation and re-epithelialization via mechanotransductive signaling pathways [16]. Multiple randomized controlled trials and meta-analyses have demonstrated the superiority of NPWT relative to conventional moist wound care in accelerating wound closure, particularly within diabetic populations [17,18]. Consistent with these findings, our data indicate that NPWT was predominantly administered to patients with CN, whose wounds exhibited greater chronicity and neuropathic etiology. The observed gradual yet progressive healing trajectory in CN wounds likely reflects NPWT’s capacity to address deep tissue defects and mitigate edema associated with neuropathic pathology [19].

Conversely, ozone therapy confers a complementary biochemical modality characterized by potent antimicrobial, anti-inflammatory, and tissue oxygenation-enhancing effects. Ozone induces controlled oxidative stress that activates endogenous antioxidant defenses, promotes vasodilation, and stimulates angiogenesis, thereby augmenting tissue oxygen tension essential for reparative processes [20,21]. Our findings highlight the preferential application of ozonated oil dressings in DFS patients, who typically present with acute, infection-prone ulcers. Marked reductions in infection biomarkers, including procalcitonin (PCT) and C-reactive protein (CRP), coupled with enhanced granulation tissue formation, corroborate ozone therapy’s efficacy in microbial eradication and tissue regeneration [20,22].

Biochemical analyses reveal divergent profiles between DFS and CN cohorts, reflecting their underlying pathophysiologies. DFS patients manifested elevated inflammatory and infectious markers such as PCT and hemoglobin, indicative of an acute bacterial inflammatory state characteristic of diabetic foot infections [23]. In contrast, CN patients exhibited elevated D-dimer levels and diminished transcutaneous oxygen pressure (tcpO₂), signifying persistent inflammation, microvascular compromise, and impaired oxygen delivery, which collectively contribute to ulcer chronicity and protracted healing [24,25]. The protracted duration of CN ulcers, frequently exceeding one year, underscores the imperative for therapeutic strategies targeting neuropathic and ischemic dysfunction, exemplified by NPWT.

Microbiological assessment confirmed polymicrobial colonization across both groups, with predominant pathogens including Pseudomonas aeruginosa, *Staphylococcus aureus*, and *Enterococcus faecalis*, consistent with previously characterized diabetic wound microbiota [26]. Notably, concomitant systemic antibiotic administration and topical ozone therapy in DFS patients facilitated effective infection control, as evidenced by reestablishment of physiological flora and significant declines in infection biomarkers after six weeks of treatment. This synergistic effect suggests ozone therapy potentiates antibiotic efficacy through its broad-spectrum antimicrobial and immunomodulatory properties [13,20].

A pivotal finding of this study, supported by emerging evidence, is the potential synergism of combined NPWT and ozone therapy. This integrated approach leverages NPWT’s mechanical optimization of the wound environment and edema reduction alongside ozone’s antimicrobial and pro-angiogenic mechanisms, culminating in enhanced capillary density, improved tissue oxygenation, and accelerated wound contraction [27]. Our clinical outcomes align with those reported by Banasiewicz et al., demonstrating superior healing trajectories and microcirculatory improvements through this dual modality [27]. Such synergy is particularly salient in complex, chronic wounds such as CN ulcers, where multifactorial impediments to healing coexist.

Despite these encouraging outcomes, challenges remain in standardizing and clinically validating ozone therapy. Whereas NPWT efficacy is substantiated by robust randomized controlled trials and meta-analyses [17,18], the evidence base for ozone therapy primarily comprises smaller, heterogeneous studies with variable dosing protocols and application methods, complicating direct comparison and guideline development [20,21]. Our data emphasize the need for rigorously designed, adequately powered multicenter randomized trials to optimize ozone therapy regimens and elucidate its additive benefit in conjunction with NPWT.

Beyond NPWT and ozone therapy, numerous adjunctive therapeutic modalities have been explored for chronic, refractory wounds. These may be employed individually or in combination to enhance healing outcomes. Prominent among these are stem cell therapies and growth factor applications, which potentiate tissue regeneration by activating intrinsic reparative cellular mechanisms [28]. Hyperbaric oxygen therapy (HBOT), involving administration of oxygen at increased atmospheric pressures, has demonstrated efficacy in enhancing oxygen delivery to hypoxic wound tissues, thereby accelerating reparative processes [29]. Biological dressings comprising living cells, extracellular matrix components, or synthetic polymers provide structural support and modulate the wound microenvironment to promote healing [30].

Physical adjuncts such as ultrasound and low-level laser therapies improve microcirculation and modulate inflammatory responses, facilitating tissue repair [31,32]. Enzymatic and chemical debridement agents serve to remove necrotic tissue and disrupt bacterial biofilms, preparing the wound bed for optimal healing [33,34]. Additionally, targeted pharmacological interventions aimed at infection control, inflammation modulation, and enhancement of local perfusion—via vasodilators and anti-inflammatory agents—remain foundational elements of comprehensive wound management [35,36]. Collectively, these modalities underscore the necessity for a multimodal, individualized approach to managing chronic wounds that addresses the complex pathophysiological factors impairing healing.

## 5. Conclusions

In conclusion, this study elucidates the distinct clinical and biochemical profiles of DFS and CN, underscoring the need for tailored therapeutic strategies. NPWT remains a cornerstone for chronic neuropathic wounds, while ozone therapy serves as a valuable adjunct targeting infection and ischemia in acute diabetic foot ulcers. The combined application of these therapies, complemented by emerging alternative modalities, constitutes a promising multimodal paradigm that addresses both mechanical and biochemical barriers to wound repair. Future research should prioritize the development of evidence-based clinical guidelines through large-scale, controlled trials to optimize therapeutic outcomes for patients suffering from complex diabetic foot pathologies.

## Figures and Tables

**Table 1:** General characteristics of patients with diabetic foot syndrome and Charcot neuroarthropathy.

	DFS *n* = 30	CN *n* = 30	*p*
Sex; *n* (%)			
Women	6 (20.00)	9 (30.00)	0.551
Men	24 (80.00)	21 (70.00)	
Age; mean ± SD	67.13 ± 12.05	66.23 ± 14.76	0.797
Age; *n* (%)			
<50	3 (10.00)	7 (23.33)	0.398
50–59	4 (13.33)	1 (3.33)	
60–69	7 (23.33)	6 (20.00)	
70–79	12 (40.00)	10 (33.33)	
≥80	4 (13.33)	6 (20.00)	
Body weight (kg); mean ± SD	90.33 ± 16.53	85.33 ± 18.57	0.275
Height (cm); mean ± SD	177.53 ± 9.57	175.23 ± 11.98	0.415
BMI (kg/m^2^); mean ± SD	28.56 ± 4.18	27.77 ± 5.16	0.516
BMI; *n* (%)			
<18.5 underweight	0 (0.00)	0 (0.00)	0.517
18.5–24.99 proper weight	8 (26.67)	12 (40.00)	
25.0–29.99 overweight	11 (36.67)	8 (26.67)	
≥30.0 obesity	11 (36.67)	10 (33.33)	
Using of stimulants; *n* (%)			
Alcohol	5 (16.67)	5 (16.67)	1
Cigarette	13 (43.33)	9 (30.00)	0.422
Diabetes; *n* (%)			
Type 1	25 (83.33)	22 (73.33)	0.531
Type 2	5 (16.67)	8 (26.67)	
Mobility capacity; *n* (%)			
Correct	0 (0.00)	1 (3.33)	
Restricted	30 (100.00)	29 (96.67)	
Assistance during moving; *n* (%)			
Crutches	25 (83.33)	22 (73.33)	0.531
Walker	3 (10.00)	2 (6.67)	1
Wheelchairs	6 (20.00)	7 (23.33)	1
Off-loading footwear	20 (66.67)	0 (0.00)	-

**Table 2:** Comparison of laboratory results of patients with diabetic foot syndrome and Charcot neuroarthropathy.

Parameter	DFS *n* = 30	CN *n* = 30	*p*
CRP (mg/L)	195.50 [110.00; 280.00]	122.50 [80.00; 220.00]	0.087
PCT (%)	0.86 [0.64; 0.96]	0.61 [0.46; 0.76]	**0.003**
Glycated hemoglobin (%)	7.20 [6.70; 8.45]	7.20 [6.40; 8.40]	0.689
HGB morphology (g/dL)	12.59 ± 1.89	11.09 ± 1.46	**0.001**
D-dimers (µg/L)	400.00 [280.00; 470.00]	484.00 [420.00; 500.00]	**0.004**
Fibrinogen (mg/dL)	320.04 ± 119.98	367.41 ± 165.19	0.300
APTT (s)	36.00 [36.00; 36.00]	36.00 [32.70; 36.00]	0.754
Protein C activity (%)	130.00 [100.00; 140.00]	120.00 [120.00; 130.00]	0.711
Total protein (g/L)	90.00 [81.00; 90.00]	90.00 [80.00; 90.00]	0.777
Na (mmol/L)	136.00 [134.00; 136.00]	135.50 [134.00; 138.00]	0.556
K (mmol/L)	4.46 ± 0.38	4.71 ± 0.57	0.051
Uric acid (mg/dL)	4.00 [3.00; 5.00]	5.00 [4.00; 6.00]	0.064

*p* < 0.05 as statistically significant.

**Table 3:** Comparison of CRP and PCT results after 3 and 6 weeks of treatment in the DFS and CN groups.

Time of Treatment	Markers of Inflammation	DFS *n* = 30	CN *n* = 30	*p*
0 week	CRP (mg/L)	195.50 [110.00; 280.00]	122.50 [80.00; 220.00]	0.087
	PCT (%)	0.86 [0.64; 0.96]	0.61 [0.46; 0.76]	**0.003**
After 3 weeks	CRP (mg/L)	50.00 [20.00; 75.00]	45.00 [12.50; 99.00]	0.785
	PCT (%)	0.46 (0.40; 0.60)	0.38 (0.36; 0.45)	**0.003**
After 6 weeks	CRP (mg/L)	8.00 [5.00; 10.00]	5.00 [5.00; 6.00]	**0.002**
	PCT (%)	0.36 [0.36; 0.39]	0.36 [0.20; 0.36]	**0.015**

*p* < 0.05 as statistically significant.

**Table 4:** Frequency of therapies used in patients with diabetic foot syndrome and Charcot neuroarthropathy.

Therapy	DFS *n* (%) *n* = 30	CN *n* (%) *n* = 30	*p*
Ozonated saline at a concentration of 70 μg/mL	30 (100.00)	29 (96.67)	1
Ozonated shoe at a concentration of 70 µg/mL	29 (96.67)	30 (100.00)	1
Synthetic bone graft substitute (Beta-TCP)	3 (10.00)	5 (16.67)	0.704
Special antiseptic dressing (ozonated olive oil)	30 (100.00)	15 (50.00)	**<0.001**
Negative pressure wound therapy (NPWT)	14 (46.67)	21 (70.00)	0.116

*p* < 0.05 as statistically significant.

**Table 5:** Assessment of the blood vessels’ condition.

Variable	DFS *n* = 30	CN *n* = 30	*p*
Ankle–brachial index (ABI)	1.00 [0.98; 1.00]	1.00 [0.98; 1.00]	0.176
Transcutaneous oxygen tension (tcpO_2_). (mmHg)	45.00 [35.00; 60.00]	35.00 [30.00; 50.00]	**0.018**
Arterial ischaemia. *n* (%)			
Major	0 (0.0)	1 (3.33)	1
Moderate	4 (13.33)	3 (10.00)	1
None	26 (86.67)	21 (70.00)	0.21
Thrombosis	5 (16.67)	8 (26.67)	0.531

*p* < 0.05 as statistically significant.

**Table 6:** Comparison of wound size at three time points.

Variable	DFS *n* = 30	CN *n* = 30	*p*
Baseline wound size (cm^3^), median (IQR)	5.00 (2.00. 32.00)	8.00 (2.40; 19.20)	0.641
Wound size at 3 weeks (cm^3^), median (IQR)	0.40 (0.15; 2.00)	1.50 (0.32; 4.00)	0.056
Wound size at 6 weeks (cm^3^), median (IQR)	0.002 (0.001; 0.0025)	0.004 (0.001; 0.05)	0.066
Difference at 3 weeks (cm^3^), median (IQR)	−4.425 (−17.00; −1.95)	−4.450 (−12.80; −1.90)	0.959
Wound reduction at 3 weeks (%), median (IQR)	92.71 (75.00–96.25)	76.98 (62.50–91.11)	**0.005**
Difference at 6 weeks (cm^3^), median (IQR)	−4.99 (−31.75; −2.00)	−7.75 (−19.18;−2.25)	0.657
Wound reduction at 6 weeks (%), median (IQR)	99.98 (99.90; 99.99)	99.90 (98.96; 99.98)	0.055

*p* < 0.05 as statistically significant.

**Table 7:** Assessment of ulceration in patients with diabetic foot syndrome and Charcot neuroarthropathy.

Variable	DFS *n* = 30	CN *n* = 30	*p*
Duration of ulceration, *n* (%)			
A year or more	8 (26.67)	20 (66.67)	**0.007**
A month or more	21 (70.00)	10 (33.33)	
A week or more	1 (3.33)	0 (0.00)	
A few days	0 (0.00)	0 (0.00)	
Time of previous treatment, *n* (%)			
A year or more	1 (3.33)	11 (36.67)	**0.007**
A month or more	18 (60.00)	15 (50.00)	
A week or more	10 (33.33)	4 (13.33)	
A few days	1 (3.33)	0 (0.00)	
Wound location, *n* (%)			
sole of the foot	14 (46.67)	30 (100.00)	**<0.001**
Forefoot	7 (23.33)	6 (20.00)	1
Toe I	18 (60.00)	0 (0.00)	**<0.001**
Toes II, III, IV, and V	17 (56.67)	0 (0.00)	**<0.001**
Other characteristics, *n* (%)			
No pain or mild pain	1 (3.33)	5 (16.67)	0.197
Sensory disorders	30 (100.00)	28 (93.33)	0.472
The foot is properly insulated and pink	20 (66.67)	4 (13.33)	**<0.001**
Palpable pulse in the foot	30 (100.00)	22 (73.33)	**0.008**

*p* < 0.05 as statistically significant.

**Table 8:** Comparison of symptoms of infection at the time of starting treatment.

Wound Infection Symptoms	DFS *n* = 30	CN *n* = 30	*p*
Local			
Increased pain	27 (90.00)	19 (63.33)	**0.033**
Erythema	25 (83.33)	10 (33.33)	**<0.001**
Swelling	26 (86.67)	21 (70.00)	0.21
Locally elevated temperature	13 (43.33)	9 (30.00)	0.422
Increased exudate	22 (73.33)	17 (56.67)	0.279
Delayed healing	6 (20.00)	7 (23.33)	1
Granulation tissue prone to rupture	3 (10.00)	3 (10.00)	1
Fistulas	28 (93.33)	23 (76.67)	0.148
Granulation tissue with punctate biofilm	28 (93.33)	21 (70.00)	**0.045**
Granulation tissue with punctate fibrin	1 (3.33)	5 (16.67)	0.197
Spreading/Systemic			
Intense erythema	8 (26.67)	6 (20.00)	0.76
Fever	7 (23.33)	2 (6.67)	0.148
Abscess/pus	24 (80.00)	11 (36.67)	**0.002**
Cellulitis	13 (43.33)	16 (53.33)	0.605
General weakness	16 (53.33)	9 (30.00)	0.116
Increased white blood cell count	4 (13.33)	2 (6.67)	0.667
Osteitis	29 (96.67)	17 (56.67)	**0.001**
Bone fragments	9 (30.00)	17 (56.67)	0.068

*p* < 0.05 as statistically significant.

## Data Availability

The original contributions presented in this study are included in the article. Further inquiries can be directed to the corresponding author.
